# Incident stroke among Ghanaians with hypertension and diabetes: A multicenter, prospective cohort study

**DOI:** 10.1016/j.jns.2018.09.018

**Published:** 2018-12-15

**Authors:** Fred S. Sarfo, Linda M. Mobula, Jacob Plange-Rhule, Daniel Ansong, David Ofori-Adjei

**Affiliations:** a Department of Medicine, Kwame Nkrumah University of Science & Technology, Kumasi, Ghana; b Department of Medicine, Komfo Anokye Teaching Hospital, Kumasi, Ghana; c Johns Hopkins University School of Medicine, Baltimore, MD, USA; d Johns Hopkins University Bloomberg School of Public Health, Baltimore, MD, USA; e Ghana College of Physicians and Surgeons, Accra, Ghana; f Department of Medicine & Therapeutics, University of Ghana School of Medicine and Dentistry, Accra, Ghana

**Keywords:** Incident stroke, Risk factors, Prospective study, West Africa

## Abstract

**Background:**

The burden of stroke among hypertensive and diabetic population in sub-Saharan Africa remains high. We sought to identify the risk factors associated with stroke occurrence in these high-risk population groups.

**Methods:**

A prospective cohort study involving adults with hypertension and or type II diabetes mellitus at 5 public hospitals in Ghana who were stroke-free at enrollment. Patients were followed every 2 months at clinic for 18 months and assessed clinically for first ever stroke by physicians. We calculated crude incidence rates for stroke and assessed the factors associated with stroke occurrence using a multivariate Cox Proportional Hazards regression models.

**Results:**

Of 3220 eligible participants with 3805 person-years of follow-up, there were 54 clinically confirmed new strokes. Incidence rate of stroke was 14.19 events per 1000 person-years [95% CI: 10.77–18.38], with rates among diabetics with hypertension being 16.64 [10.58–25.00], hypertension of 13.77 [9.33–19.64] and diabetes was 9.81 [3.59–21.74]. Two factors independently associated with stroke occurrence were previous cigarette smoking with adjusted HR (95% CI) of 2.59 (1.18–5.67) and physical inactivity, 1.81 (1.06–3.10). In secondary analysis, stage II hypertension compared with optimal BP was associated with aHR of 3.04 (1.00–9.27), *p* = .05 for stroke occurrence.

**Conclusion:**

Incident stroke among Ghanaians with hypertension and diabetes is quite high. Stricter control of blood pressure and engaging in regular physical activities are strongly recommended to reduce the risk of strokes.

## Highlights

• The first prospective cohort study to assess factors associated with incident strokes among Ghanaians
• 3220 participants stroke free adults with hypertension or diabetes were followed for an average of 14 months
• There were 54 strokes with incidence rate of 14.19 events per 1000 person-years
• Patients with both diabetes with hypertension had highest stroke rates
• Previous cigarette smoking and physical inactivity were independently associated with incident strokes

## 1 Introduction

Recent estimates indicate that sub-Saharan Africa (SSA) currently bear a high and rising burden of stroke on the globe [[1], [2], [3], [4], [5], [6], [7]]. Stroke in these regions is characterized by young age of onset, a high propensity of being hemorrhagic, a high mortality, and significant post-stroke complications including depression, cognitive impairment and social stigma [[8], [9], [10], [11], [12], [13], [14], [15], [16], [17], [18], [19]]. Due to the pervasive lack of health personnel and weak health infrastructure to support stroke survivors in these regions, there is a dire need to identify the key risk factors for stroke occurrence to inform interventions aimed at stroke prevention at the population level.

The INTERSTROKE [11] and GBD studies [10] which included African participants provided some insights into the potential risk factors for stroke occurrence in SSA. The Stroke Investigative Research and Educational Networks (SIREN), the largest case-control study on stroke in Africa [12] to date have recently identified and characterized the associations between 11 dominant risk factors of stroke in decreasing order of population attributable risk as hypertension, dyslipidemia, regular meat consumption, elevated waist-to-hip ratio, diabetes mellitus, low consumption of green leafy vegetables, stress, table added salt, cardiac disease, physical inactivity and current cigarette smoking [12]. These cardio-metabolic and lifestyle risk factors associated with stroke occurrence provide a clear evidence of the impact of the epidemiologic transition, driven by rapid urbanization and adoption of westernized lifestyles, on the surge of Cardiovascular Diseases (CVD) among Africans.

Prospective studies aimed at identifying risk factors for stroke occurrence among indigenous Africans are also urgently warranted. While case-control studies can identify risk factors associated with stroke occurrence, causal inferences cannot be drawn. The prospective, community-based Tanzania Stroke Incidence Project (TSIP) study reported a yearly stroke incidence rate of 108.6 per 100,000 in rural Hai and 315.9 per 100,000 in urban Dar-es-Sallam [4]. Among the factors, the TSIP investigators identified as associated with stroke occurrence were previous cardiac event, HIV infection, high ratio of total cholesterol to HDL cholesterol, smoking and hypertension [20]. An emerging view supported by recent secular trends is that the growing burden of stroke in SSA is driven by a neglect in control of vascular risk factors such as hypertension and diabetes at the population level. In this regard, prospective data on stroke risk among high risk population such as those with hypertension and diabetes are needed to elucidate the key contributors to stroke occurrence to fine-tune primary prevention interventions. We therefore sought to evaluate the determinants of stroke among a prospective cohort Ghanaians with hypertension and diabetes mellitus. Participants were recruited as part of a pragmatic clinical trial aimed at improving access to medicines for the control of hypertension and diabetes by offering medications at differential pricing [21].

## 2 Methods

### 2.1 Study design and participants

The Ghana Access and Affordability Program (GAAP) pilot study is a prospective cohort study involving adults with hypertension only (HPT), hypertension with diabetes mellitus (HPT + DM) and diabetes mellitus only (DM) at public hospitals in Ghana. Participants were recruited from five study sites including the Agogo Presbyterian Hospital, (APH), Atua Government Hospital, (AGH), Komfo Anokye Teaching Hospital, (KATH), Kings Medical center, (KMC) and the Tamale Teaching Hospital, (TTH). Ethical approval was obtained from all study sites. The study protocol has been published elsewhere [21].

### 2.2 Evaluation of study participants

Trained research assistants obtained informed consent before participants were enrolled into the study. Demographic information including age, gender, educational attainment, employment status, and lifestyle behaviors such as alcohol use, cigarette smoking, level of physical activities, frequency and daily quantities of fruits and vegetable consumption as well as table added salt were assessed through interviews and responses collected on a questionnaire. A detailed medical history including duration of hypertension or diabetes diagnosis and doses of medications currently being taken were obtained. Anthropometric evaluations including measurement of weight, height and waist circumference were performed by Study nurses. Body mass index (BMI) of each participant was then derived by dividing the weight in kilograms by the square of the height in meters.

### 2.3 Laboratory measurements

To ensure standardization across all study sites, an International Organization for Standardization (ISO)-certified and quality-assured laboratory was contracted to run all biochemical panels which included serum creatinine, lipid profile and hemoglobin A1C for subjects with diabetes. Samples were transported to the laboratory by trained phlebotomists on the same day of collection often within 4 h or where not feasible (KMC and AGH sites), samples were stored in a freezer before transported to the laboratory the next day.

### 2.4 Stroke diagnosis

Stroke diagnosis was based on the World Health Organization definition [22], if participant had ever experienced sudden onset of weakness or sensory loss on one side of the body, sudden loss of vision, or sudden loss of speech. These questions were obtained from the 8-item questionnaire for verifying stroke free status (QVSFS) which has been validated locally [[23], [24], [25]]. QVSFS was used as neuro-imaging facilities were not available at any of the study sites at the time of the study. Study participants visited every 2 months for 18 months to assess control of hypertension and diabetes mellitus and to assess for vascular complications including stroke.

Individuals were classified as physically active if they were regularly involved in moderate exercise (walking, cycling, or gardening) or strenuous exercise (jogging, football, and vigorous swimming) for 4 h or more per week. Alcohol use was categorized into current users (users of any form of alcoholic drinks) or never/former drinker while alcohol intake was categorized as low drinkers (1–2 drinks per day for female and 1–3 drinks per day for male) and high drinker (>2 drinks per day for female and > 3 drinks per day for male. 1 drink or 1 unit of alcohol = 8 g of alcohol) [26]. Smoking status was defined as current smoker (individuals who smoked any tobacco in the past 12 months) or never or former smoker [26]. Vegetable and fruit intake was assessed based on number of daily servings per week. The Ghana Statistical Services defines urban residence as settlements with population > 20,000, peri-urban as settlements with population size between 5000 and 19,999 and rural residence as those with population < 5000.

### 2.5 Statistical analysis

The main outcome measure was time to onset of new stroke during 18 months of prospective follow-up. Patients with a prior history of stroke before study onset were excluded from the present analyses. For baseline characteristics, means were compared using the Student's *t*-test for 2-group comparisons and proportions were compared using the Chi-squared tests or Fisher's exact test for proportions with subgroupings <5. Crude incidence rates were calculated and expressed as events/1000-person years of follow-up and 95%CI calculated using the Mid-P exact test. A multivariate Cox Hazards Proportion regression analysis was fitted to identify factors independently associated with the risk of stroke. Patients were censored either at the date of stroke diagnosis, at the last visit for those who were lost-to-follow up, or at July 31, 2017 for the remainder. Independent variables evaluated included the following *socio-demographic factors*: age, gender, location of residence, employment status, *lifestyle/behavioral factors*: included previous cigarette smoking, current alcohol use, physical activity, table added salt, fruit and vegetable intake; *health system factors:* level of healthcare institution (primary, secondary or tertiary), *patho-biologic factors:* central obesity, duration of hypertension or diabetes, number of antihypertensive medications, and baseline systolic and diastolic BP as well as baseline HBA1C. Variable selection was based on clinical and empirical significance of covariates in the model. Variables were included in the multivariate analyses upon meeting a *p*-value cut-off of <0.05 in bivariate unadjusted regression analysis. In all analyses, two-tailed *p*-values <.05 were considered statistically significant. Secondary analyses included the determinants of stroke among participants with any hypertension (HPT or DM/HPT) or diabetes (DM + DM/HPT). Model diagnosis and fit were assessed using residual plots analysis and visual inspection for collinearity of variables in the Cox models. Statistical analysis was performed using Graphpad Prism version 7 and SPSS version 20.

## 3 Results

### 3.1 Demographic, lifestyle and clinical characteristics of cohort

Between July 1, 2015 and April 30, 2016, we enrolled 3296 participants comprising of 2520 (76.5%) females. Follow-up was completed on July 31, 2017. At enrollment, 1867 (56.7%) had hypertension only (HTN only), 1006 (30.5%) had both hypertension and diabetes (HTN + DM) and 422 (12.8%) had diabetes (DM only). At enrollment, 76 (2.3%) had clinically confirmed strokes and were excluded from further analysis in the prospective cohort for this report. Of the 3220 eligible participants with 3805 person-years of follow-up, there were 54 clinically confirmed new strokes. The mean duration of follow-up per participant was 14.2 ± 5.9 months with 1806 (56.1%) completing month 18 visit.

Participants who suffered a stroke during follow-up were more likely to be male and were significantly older, with a mean ± SD age of 61.4 ± 10.6 years compared with 57.4 ± 12.8 years for those stroke-free, *p* = .02. There were significant differences between the two groups with regards to the level of health institution with higher rates reported at tertiary and primary levels compared with secondary level. Furthermore, those who experienced strokes were significantly more likely to be unemployed, 46.2% versus 31.3% among those who did not experience stroke, *p* = .02. Educational status, monthly income, health expenditures and location of residence did not significantly differ between the two groups (Table 1).

**Table 1:** Demographic and clinical characteristics of participants with incident stroke versus those without stroke.

	Stroke, *N* = 54	No stroke, *n* = 3166	P-value
Age, mean ± SD	61.4 ± 10.6	57.4 ± 12.8	**0.02**
Male gender, n (%)	20 (37.0)	721 (22.8)	**0.01**
Disease class, n (%)			0.36
Hypertension only	28 (51.9)	1786 (56.4)	
Type 2 Diabetes Mellitus only	5 (9.3)	414 (13.1)	
Both Hypertension and T2DM	21 (38.9)	966 (30.5)	
Level of institution, n (%)			**0.02**
Tertiary	40 (74.1)	1813 (57.3)	
Secondary	10 (18.5)	1184 (37.4)	
Primary	4 (7.4)	169 (5.3)	
Location of residence, n (%)			0.19
Urban	27 (50.0)	1377 (43.5)	
Peri-urban	15 (27.8)	713 (22.5)	
Rural	12 (22.2)	1076 (34.0)	
Educational attainment, n (%)			0.65
Tertiary	6 (11.1)	355 (11.2)	
Secondary	19 (35.2)	1107 (35.0)	
Primary	12 (22.2)	516 (16.3)	
None	17 (31.5)	1187 (37.5)	
Unemployed, n (%)	25 (46.2)	992 (31.3)	**0.02**
National Health Insurance cover for all medicines, n (%)	25 (46.2)	1498 (47.3)	0.88
Monthly Income levels, n (%)			0.90
> 1000 GHS	3 (5.6)	250 (7.9)	
210–1000 GHS	14 (25.9)	848 (26.8)	
< 210 GHS	20 (37.0)	1173 (37.1)	
Unknown	17 (31.5)	894 (28.2)	
Expenditures on medications, Mean ± SD (GHS)	16.8 ± 29.0	21.6 ± 48.8	0.47
Cigarette use			**0.001**
Current use	0 (0.0)	16 (0.5)	
Former use	11 (20.4)	198 (6.3)	
Never use	43 (79.6)	2952 (93.2)	
Current alcohol use, n (%)	4 (7.4)	240 (7.6)	0.96
Table added salt, n (%)	7 (13.0)	591 (18.7)	0.29
Physical inactivity, n (%)	27 (50.0)	1205 (38.1)	0.07
Fruit intake: daily servings/week, mean ± SD	2.2 ± 1.7	2.6 ± 2.1	0.15
Fruit intake daily servings/week, n (%)			0.09
0	8 (14.8)	463 (14.6)	
1 to 3	38 (70.4)	1819 (57.5)	
4 to 7	8 (14.8)	880 (27.8)	
Vegetable intake: daily servings/week, mean ±	4.8 ± 2.1	4.9 ± 2.2	0.76
Vegetable intake, n (%)			**0.04**
1 to 2	17 (34.0)	568 (18.0)	
> 3	43 (66.0)	2592 (82.0)	
Heart failure, n (%)	4 (7.4)	174 (5.5)	0.54
Body mass index, mean ± SD kg/m^2^	26.0 ± 4.8	26.6 ± 5.6	0.50
Raised waist circumference, n (%)	27 (50.0)	1935 (61.1)	0.10
Duration of Hypertension (years)			
mean ± SD	9.1 ± 6.7	7.8 ± 7.2	0.22
median (IQR)	8 (4–14)	6 (3−10)	**0.05**
Duration of Diabetes (years)			
mean ± SD	10.1 ± 5.8	9.2 ± 7.0	0.52
median (IQR)	11 (4.8–15.3)	8 (4–13)	0.24
Antihypertensive medications, mean ± SD	2.1 ± 1.2	1.8 ± 1.0	**0.05**
Anti-hypertensive medications	2.3 ± 0.63	2.2 ± 0.68	0.3
ACE-I	27 (50.0)	1284 (40.6)	0.16
ARB	16 (29.6)	806 (25.5)	0.49
Beta blocker	5 (9.3)	269 (8.5)	0.84
Calcium channel blocker	43 (79.6)	2121 (67.0)	0.05
Diuretics	11 (20.4)	857 (27.1)	0.27
Methyl dopa	10 (18.5)	427 (13.5)	0.24
Hydrallazine	2 (3.7)	41 (1.3)	0.13
Statin	7 (13.0)	313 (9.9)	0.45
Aspirin	8 (14.8)	317 (10.0)	0.25
eGFR, (ml/min) mean ± SD	67.4 ± 21.0	76.9 ± 16.0	<0.0001
HBA1C, mean ± SD	9.0 ± 2.9	8.6 ± 2.6	0.54

ACE-I = Angiotensin-converting enzyme inhibitor; ARB = Angiotensin Receptor Blocker, eGFR = estimated glomerular filtration rate derived using the CKD-EPI formula from serum creatinine measurement (data available for 2631 participants).

Bold indicates p-values less than 0.05.

Among the lifestyle factors, previous cigarette smoking history was more frequently reported among stroke group at 20.4% compared with 6.3% among the stroke-free group, *p* = .0001. Physical inactivity rates were marginally higher among stroke group at 50.0% compared with 38.1% among those without stroke, *p* = .07. A higher proportion of those who were stroke free consumed more daily servings of vegetables and fruits during the week compared with those with stroke. Current alcohol use and table added salt were not significantly different between the two groups.

### 3.2 Incidence rates of stroke

**Fig. 1:**
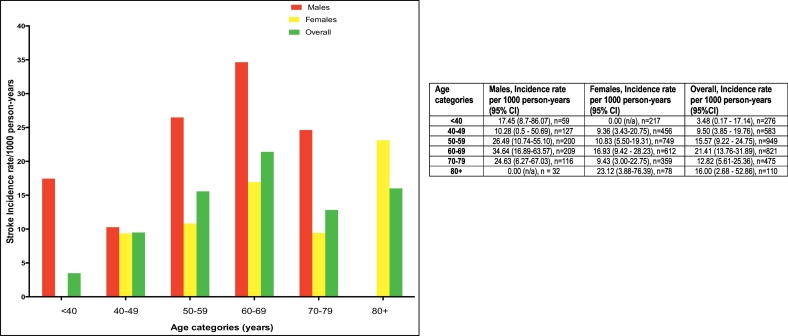
Incidence rates of stroke by age groups and gender. Table insert showing incidence rates with 95% CI by age and gender.

**Fig. 2:**
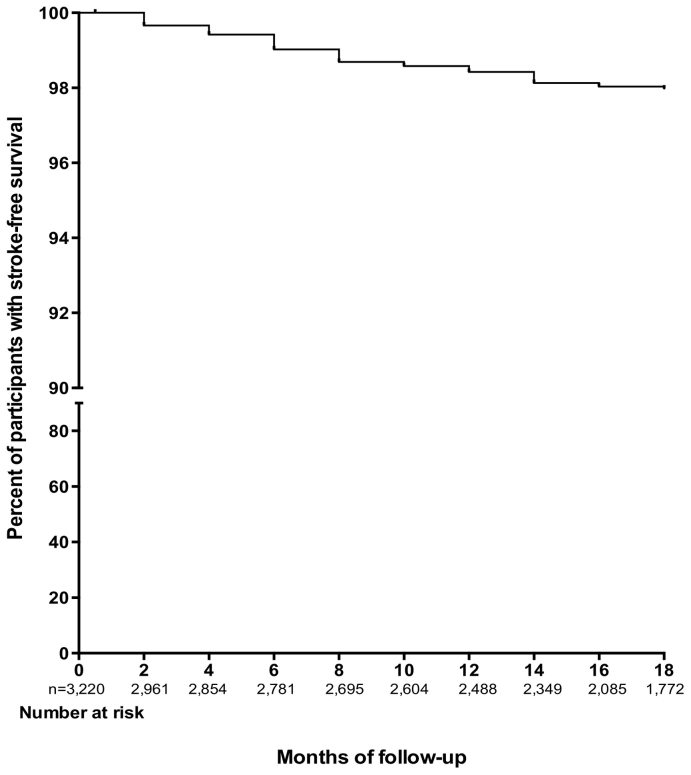
Kaplan Meier plot showing the proportion of study participants with stroke free survival.

Incidence rate of stroke overall was 14.19 events per 1000 person-years [95% CI: 10.77–18.38]. Incidence rate among age < 40 years was 3.48, 40–49 years was 9.50, 50–59 years was 15.57, 60–69 years was 21.41, 70–79 years was 12.82 and 80+ years was 16.00 per 1000-person years. Fig. 1 depicts incidence rate by gender and age categories. There were 5 strokes among 419 diabetics, 21 out of 985 diabetics with hypertension had strokes and 28 strokes among 1816 participants with hypertension. Incidence rates of stroke among those with diabetes alone was 9.81 (3.59–21.74)/1000 person-years, hypertension alone was 13.77 (9.33–19.64)/1000 person-years, and among those with both diabetes with hypertension was 16.64 (10.58–25.00)/1000 person-years. Probability of stroke-free survival at month 18 for the overall cohort was 98.0% (Fig. 2), being 98.2% for participants with diabetes, 97.2% for those with hypertension and 97.0% for those with both diabetes and hypertension.

### 3.3 Risk factors for stroke occurrence among the cohort

Six (6) factors associated with stroke occurrence in unadjusted analysis included male gender, increasing age, receiving care at a secondary level health facility, being unemployed, previous cigarette smoking, and physical inactivity. Two factors independently associated with stroke occurrence were previous cigarette smoking with adjusted HR (95% CI) of 2.59 (1.18–5.67) and physical inactivity, 1.81 (1.06–3.10). Table 2. Duration of cigarette use before cessation was significantly associated with stroke risk with HR of 1.28 (95% CI: 1.07–1.53) for every 5-year increase in cigarette use before cessation. We did not collect data on duration of cigarette cessation before entry into the study. Current cigarette use was not associated with stroke occurrence in unadjusted analysis, HR of 0.00 (0.00–2.59). There was however no dose-dependent relationship between duration of daily physical activity and stroke risk.

**Table 2:** Predictors of incident stroke among Ghanaians with hypertension and/or diabetes.

Predictors	Unadjusted HR	P-value	Adjusted HR	P-value
Male gender	2.10 (1.21–3.64)	**0.009**	1.15 (0.89–1.48)	0.16
Age/10 year increase	1.28 (1.03–1.60)	**0.03**	1.59 (0.83–3.06)	0.28
Level of Health Facility				
Tertiary level	0.85 (0.30–2.38)	0.76	1.05 (0.62–1.78)	0.85
Secondary level	0.30 (0.09–0.94)	0.04	0.44 (0.13–1.51)	
Primary level	1.00		1.00	
Disease class				
Hypertension only	1.38 (0.53–3.58)	0.51	–	
Hypertension and Type 2DM	1.72 (0.65–4.56)	0.28	–	
Type 2 DM	1.00		–	
Unemployed	1.89 (1.11–3.23)	**0.02**	1.52 (0.83–2.78)	0.18
Previous Cigarette smoking	3.68 (1.90–7.14)	**0.0001**	2.59 (1.18–5.67)	**0.02**
Physical inactivity	1.71 (1.00–2.92)	**0.05**	1.81 (1.06–3.10)	**0.03**
Vegetable intake	0.97 (0.86–1.09)	0.63	–	
1 to 2	1.21 (0.62–2.35)	0.57	–	
>2	1.00			
Fruit intake	0.90 (0.78–1.04)	0.16	–	
Location				
Urban	1.36 (0.97–1.91)	0.08	–	
Semi-urban	1.95 (0.91–4.18)	0.08	–	
Rural	1.00		–	

Bold indicates p-values less than 0.05.

Among participants with any hypertension (hypertension only and hypertension with Type 2 DM), male gender, increasing age, unemployed, previous cigarette smoking and location of residence were associated with stroke occurrence in unadjusted analyses. In addition to previous cigarette smoking, residence in an urban location was associated with an adjusted HR of 1.45 (1.00–2.12) and semi-urban location with adjusted HR of 2.39 (1.06–5.39) compared with those with residence in rural locations as a referent group, Table 3. However among participants with diabetes mellitus, only previous cigarette smoking was significantly associated with stroke occurrence in adjusted analyses, Table 4.

**Table 3:** Predictors of incident stroke among Ghanaians with hypertension and dual diagnosis of hypertension and Type 2 diabetes mellitus.

Predictors	Unadjusted HR (95% CI)	P-value	Adjusted HR (95% CI)	P-value
Male gender	2.14 (1.20–3.82)	**0.01**	1.16 (0.88–1.53)	0.29
Age/10 year increase	1.29 (1.01–1.63)	**0.04**	1.52 (0.77–3.00)	0.23
Level of Health facility				
Tertiary level	0.97 (0.58–1.63)	0.92	–	
Secondary level	0.27 (0.08–0.89)	0.03	–	
Primary level	1.00			
Disease class				
Hypertension and Type 2 DM	1.25 (0.71–2.21)	0.43		
Hypertension only	1.00			
Unemployed	1.82 (1.04–3.18)	**0.04**	1.43 (0.75–2.73)	0.27
Previous Cigarette smoking	3.72 (1.86–7.46)	**0.0002**	2.67 (1.18–6.04)	**0.02**
Physical inactivity	1.57 (0.90–2.75)	0.11	–	
Vegetable intake	0.96 (0.85–1.09)	0.56	–	
1 to 2 servings/week				
>2 servings/week				
Fruit intake	0.93 (0.80–1.07)	0.32	–	
Duration of hypertension	1.02 (0.98–1.06)	0.27	–	
Number of antihypertensives	1.21 (0.90–1.63)	0.20	–	
Location				
Urban	1.49 (1.03–2.16)	**0.03**	1.45 (1.00–2.12)	0.05
Semi-urban	2.64 (1.18–5.87)	**0.02**	2.39 (1.06–5.39)	**0.04**
Rural	1.00		1.00	

Bold indicates p-values less than 0.05.

**Table 4:** Predictors of incident stroke among Ghanaians with Type 2 diabetes mellitus and dual diagnosis of hypertension and Type 2 diabetes mellitus.

Predictors	Unadjusted HR	P-value	Adjusted HR	P-value
Male gender	1.51 (0.66–3.47)	0.33	–	
Age/10 year increase	1.25 (0.90–1.75)	0.18	–	
Level of Health facility		0.24	–	
Tertiary level	2.06 (0.62–6.85)			
Primary/Secondary level	1.00			
Disease class				
HPT + DM	1.72 (0.65–4.56)	0.28	–	
Type 2 DM only	1.00			
Unemployed	1.61 (0.74–3.48)	0.23	–	
Previous Cigarette smoking	2.76 (1.04–7.33)	0.04	2.76 (1.04–7.33)	0.04
Physical inactivity	1.05 (0.47–2.35)	0.91	–	
Vegetable intake	1.01 (0.84–1.21)	0.91	–	
1 to 2				
>2				
Fruit intake	0.88 (0.71–1.08)	0.22	–	
Duration of DM	1.01 (0.96–1.07)	0.60	–	
Number of antihypertensives	1.37 (0.95–1.99)	0.09		
Location				
Urban	1.14 (0.66–1.98)	0.64	–	
Semi-urban	1.31 (0.38–4.49)	0.66	–	
Rural	1.00			
Number of anti-diabetic medications	1.33 (0.77–2.27)	0.31	–	

### 3.4 Control of hypertension and diabetes at enrollment and stroke risk

Mean systolic blood pressure at baseline of 145.6 ± 23.0 mmHg among those subsequently developing a stroke was marginally higher than 140.9 ± 22.0 mmHg among those not developing strokes, *p* = .12. Diastolic BP was 83.6 ± 14.1 mmHg in the stroke group versus 82.0 ± 17.8 mmHg in the stroke-free group, *p* = .50. Compared with optimal BP cut-off's, patients with pre-hypertension BP had adjusted HR of 2.49 (0.86–7.22), *p* = .09, stage 1 hypertension with 1.65 (0.52–5.26), *p* = .39 and stage 2 of 3.04 (1.00–9.27), *p* = .05 upon adjustment for age, gender, employment status, physical inactivity, and cigarette smoking in secondary analysis. Hemoglobin A1C at baseline was not associated with increased stroke risk, adjusted HR of 1.07 (0.91–1.25), *p* = .41.

## 4 Discussion

This is one the few studies in SSA to characterize incident strokes among a population with hypertension and diabetes mellitus. In this multi-center, hospital-based, prospective cohort study, crude incidence rate of stroke was 14.19 per 1000 person-years. Two factors, namely physical inactivity and previous cigarette smoking were independently associated with incident stroke while 4 other factors including male gender, increasing age, seeking healthcare at a secondary level health facility and being unemployed were moderated into non-significance in adjusted models. In secondary analyses, uncontrolled baseline blood pressure, in particular grade II hypertension was independently associated with increased incident stroke risk with a hazards ratio of 3.04 (1.00–9.27) compared with optimal BP of <120/80 mmHg. However, baseline glycemic control did not independently predict stroke occurrence.

Patients with comorbid diabetes and hypertension had the highest crude incidence stroke rate of 16.64/1000 person-years, followed by hypertension with 13.77/1000 person-years and diabetes with 9.81/1000 person-years. Our stroke incidence rates among diabetics is slightly higher than that of a large Italian cohort comprising of diabetics most of whom had hypertension with almost 4 years of follow-up, where the reported incidence stroke rates was 13.7/1000 (95% CI: 7.5 to 19.8) person-years in men and 10.8/1000 (95% CI: 7.3 to 14.4) person-years in women with a history of cardiovascular disease [27]. In a sub-analysis of the North Manhattan Study, involving 1750 participants aged >60 years without DM or CKD, crude incidence rates for stroke occurrence among those with hypertension was 6.2/1000 person-years for SBP < 140 mmHg, and 10.8/1000 person-years for those with SBP > 150 mmHg [28]. Thus, although follow-up time is relatively short in the present study, the incidence rates of stroke among Ghanaian hypertensive and diabetic population appear to be slightly higher than that of developed countries.

Previous history of cigarette smoking remained a consistent determinant of stroke risk in the entire cohort and in sub-group analyses. This is an interesting observation given that the Framingham study showed that stroke risk decreased significantly 2 years after smoking cessation and was at the level of nonsmokers about 5 years after stopping cigarette smoking [29]. Mechanistically, smoking causes reduced compliance and distensibility of blood vessels and increases hematocrit, platelet aggregation and fibrogen concentrations [30]. In this Ghanaian cohort, there was dose-dependent association between duration of cigarette use before quitting and stroke risk such that each 5-year increase in cigarette smoking was associated with a 28% increased hazard of stroke. We however did not collect data on how long participants had stopped cigarette smoking before enrollment into the study. Surprisingly, current cigarette smoking was not significantly associated with increased stroke risk probably because so few of the participants reported current tobacco use. In developing countries such as ours, cigarette smoking is an expensive behavioral habit to maintain on meager incomes.

The association between physical inactivity and stroke occurrence is well established from previous case-control studies on stroke [[10], [11], [12],^31^]. In the present study, there was an 81% higher hazards of stroke among those reporting no regular physical activities compared with those with any physical activity. Although we could not establish a dose-dependent association between physical activity and stroke risk, our results suggest that lifestyle factors such physical activity level may potentiate the risk for adverse CVD outcomes among hypertensive and diabetic patients. In the prospective REGARDS (Reasons for Geographic and Racial Differences in Stroke) US cohort, physical inactivity was associated with a 20% higher hazard of stroke upon adjustment for demographic and socioeconomic factors, but further adjustment for traditional stroke risk factors attenuated this risk [32].

The level of blood pressure control at enrollment independently affected stroke occurrence in a secondary analysis. Specifically, having grade II hypertension with SBP > 160 mmHg and/or DBP > 100 mmHg was associated with a 204% higher hazards of stroke compared with optimal BP of <120/80 mmHg. The control of blood pressure across the African continent remains below 10% for those on treatment, making this an urgent public health priority [33,34]. The REGARDS investigators identified independent associations between number of antihypertensive medications prescribed for hypertensive subjects and stroke risk [35], but we did not observe such associations probably due to lower sample size, shorter duration and lack of control population without any vascular risk factor to serve as comparator. Among hypertensive patients on treatment in the present cohort, we have identified poor adherence to antihypertensive therapy, difficulties obtaining antihypertensive medications, longer duration of hypertension diagnosis, receiving healthcare at tertiary centers and number of antihypertensive medications to be independently associated with poor BP control [36]. Among Ghanaian stroke survivors with hypertension attending a neurology clinic, approximately 70% were found to have controlled BP during the first year after stroke [37]. Designing interventions aimed at improving medication adherence such as using mobile health technology [38,39] and cardiovascular polypill with generic antihypertensive medications [40,41] in resource-limited settings could lead to improvements in BP control. Furthermore, price reduction schemes for antihypertensive medications [21] and insurance coverage with task-shifting strategies [42] may be worthwhile investments in low-and-middle income countries to stem the tide of morbidity and mortality from uncontrolled hypertension.

The limitations in our study include the lack of confirmation of stroke with neuro-imaging, which is considered as the gold standard. We relied on clinical assessments by study physicians to confirm stroke diagnosis which may be subject to misclassification by stroke mimics. It was not possible to perform CT scans because of unavailability at most of the study sites which is a daunting reality in many developing countries. There is a possibility that we missed some severe or fatal stroke cases who did not report to clinic for follow-up since strokes were assessed only among participants who reported for follow-up visits. However, as part of the study protocol, participants who missed clinic visits were called to ascertain reasons for default. Well known vascular risk factors such as atrial fibrillation and dyslipidemia were not systematically assessed because electrocardiography were not undertaken for all participants and only a fourth of study population was supported by the study to cover lipid panel costs. However, we may not have been sufficiently powered for analysis of factors associated with stroke occurrence in the three subgroups because a formal power analysis was not performed *a priori.* The small number of stroke events reported overall could be due to a short follow up (average of 14 months), patients being on treatment to control blood pressure and blood glucose respectively and also significant loss to follow-up (Fig. 2). The group with dual diagnosis of hypertension and diabetes mellitus which contributed up to 39% of incident strokes were included in the models assessing factors associated with stroke occurrence among those with hypertension (Table 3) and diabetes mellitus (Table 4) respectively. In spite of these limitations, we believe our study contributes to literature by providing crude estimates of incident strokes rates and associated risk factors in a prospective cohort of hypertensive and diabetic patients. Further follow-up is planned to accrue more stroke events to enable rigorous long-term analysis of factors associated with stroke occurrence in this high-risk population.

In conclusion, incident strokes are highly common among Ghanaians with hypertension and diabetes receiving treatment in public hospitals. The situation is probably similar in other resource-limited countries in SSA. Stricter control of blood pressure and engaging in regular physical activities are strongly recommended to reduce the risk of strokes among Ghanaians with hypertension and diabetes.

## Contributors

FSS, LMM, JPR, DA and DO-A designed the study and planned analyses, and FSS wrote the first draft of the report. FSS performed statistical analyses. All authors contributed to the collection of data, discussions and interpretation of the data, and to writing of the manuscript. FSS had full access to the data. All authors reviewed and approved drafts of the report.

## Declarations

The authors do not have any competing interests.

Funding for this study was provided by MSD, Novartis, Pfizer, Sanofi (each a Participant Company) and the Bill and Melinda Gates Foundation (collectively, the Funders) through the New Venture Fund (NVF) (Investment ID: OPP1055800, Investment Title: Access and Affordability Initiative).

The NVF is a not-for-profit organization exempt as a public charity under section 501(c) (3) of the United States Internal Revenue Code of 1986, and assumes financial management of the study as a fiduciary agent and primary contractor for the Funders.

Consistent with anti-trust laws that govern industry interactions, each Participant Company independently and voluntarily will continue to develop its own marketing and pricing strategies reflecting, among other factors, the Company's product portfolios and the patients it serves. For the avoidance of doubt, the Participant Companies committed not to: (i) discuss any price or marketing strategy that may involve any Project-related product; or (ii) make any decision with respect to the presence, absence or withdrawal of any Participant Company in or from any therapeutic area; or (iii) discuss the launching, maintaining or withdrawing of any product in any market whatsoever. Each Participant Company is solely responsible for its own compliance with applicable anti-trust laws.

The Funders were kept apprised of progress in developing and implementing the study program in Ghana but had no role in study design, data collection, data analysis or in study report writing.
